# Perioperative Immunonutritional Status and Functional Recovery After Gastrectomy for Gastric Cancer: A Prospective Cohort Study of Sex-Related Differences

**DOI:** 10.3390/jcm15124558

**Published:** 2026-06-12

**Authors:** Catalin Dumitru Cosma, Vlad Olimpiu Butiurca, Marian Botoncea, Cosmin Nicolescu, Dragos Molnar, Călin Molnar

**Affiliations:** 1 Faculty of Medicine, George Emil Palade University of Medicine, Pharmacy, Sciences and Technology of Târgu Mureș, 540139 Targu Mures, Romania; catalin.cosma@umfst.ro (C.D.C.); vlad.butiurca@umfst.ro (V.O.B.); cosmin.nicolescu@umfst.ro (C.N.); dragosmolnar2000@yahoo.com (D.M.); calin.molnar@umfst.ro (C.M.); 2 General Surgery Clinic No. 1, County Emergency Clinical Hospital of Târgu-Mureș, 540136 Targu Mures, Romania

**Keywords:** gastric cancer, gastrectomy, CONUT score, immunonutrition, functional recovery, sex differences, postoperative complications, perioperative care

## Abstract

**Background:** Gastrectomy for gastric cancer is associated with substantial metabolic, nutritional, and immunological disturbances that may significantly influence postoperative recovery. Increasing evidence suggests that perioperative immunonutritional status, particularly when assessed by the Controlling Nutritional Status (CONUT) score, represents an important predictor of surgical outcomes. However, prospective data evaluating sex-related differences in postoperative nutritional recovery after gastrectomy remain limited. The aim of this study was to evaluate sex-related differences in perioperative immunonutritional status and functional recovery after gastrectomy for gastric cancer using serial CONUT score assessment. **Methods:** This prospective observational cohort study included 150 consecutive patients undergoing curative-intent gastrectomy for gastric adenocarcinoma at a tertiary referral center between 2021 and 2024. Nutritional and immune status were longitudinally assessed using the CONUT score at predefined perioperative timepoints: preoperatively (T0), early postoperatively (T1), and at 3-month follow-up (T3). Functional recovery outcomes, postoperative complications, and mid-term functional parameters were compared between male and female patients. Multivariable logistic regression analysis was performed to identify independent predictors of delayed postoperative recovery. **Results:** The study population included 91 male patients (60.7%) and 59 female patients (39.3%). Significant postoperative deterioration in albumin levels, lymphocyte counts, total cholesterol, and CONUT scores were observed in the entire cohort (*p*-time < 0.001 for all comparisons), followed by partial recovery during follow-up. No significant sex-related differences were identified regarding longitudinal immunonutritional evolution, postoperative complications, gastrointestinal recovery, or functional outcomes (*p* > 0.05). Overall postoperative complications occurred in 31.3% of patients, while 90-day mortality was 2.7%. An elevated baseline CONUT score ≥ 5 (OR 2.74, 95% CI 1.48–5.09, *p* = 0.001), postoperative CONUT score T1 ≥ 5 (OR 3.36, 95% CI 1.82–6.19, *p* < 0.001), ASA class III (OR 2.08, 95% CI 1.19–3.63, *p* = 0.010), and anastomotic leakage (OR 4.91, 95% CI 1.74–13.88, *p* = 0.003) independently predicted delayed functional recovery. Male sex was not independently associated with adverse postoperative recovery (OR 1.18, 95% CI 0.74–1.89, *p* = 0.44). **Conclusions:** Gastrectomy induces significant postoperative immunonutritional deterioration irrespective of sex. Although biological sex did not independently influence postoperative recovery trajectories, impaired perioperative immunonutritional status—particularly elevated postoperative CONUT score—was strongly associated with delayed functional recovery. Serial perioperative CONUT assessment may represent a valuable tool for individualized postoperative risk stratification and nutritional management in gastric cancer patients undergoing gastrectomy.

## 1. Introduction

Gastric cancer is a commonly encountered malignancy and its impact on health systems worldwide continues to be considerable, though its incidence is slowly declining in parts of the world. Recent estimates produced by GLOBOCAN and the Global Burden of Disease give rise to gastric cancer, already accounting for over one million new cases of cancer. In addition, there are a considerable geographical, socioeconomic, and sex differences in gastric cancer incidence and outcomes [1,2,3]. Early diagnosis initiatives, along with advancements in systemic treatment, have resulted in better prognosis in certain populations; however, surgical excision with negative margins remains an important treatment modality for gastric cancer with localized or locoregional disease [4,5,6,7]. Over the years, the surgical management of gastric cancer has improved from an oncological concept with radical excision of potentially involved tissues and lymph node stations to an extended concept that combines oncology adequacy with preservation of postoperative function and quality of life [4,5,6,7,8,9].

According to current guidelines, with a complete tumor resection there should also be adequate lymphadenectomy. The optimization of perioperative care should be done to minimize morbidity, avoid functional decline, and accelerate recovery after surgery [4,6,7,8,9]. The problem is that gastrectomy causes serious metabolic, nutritional, and immunological disorders that are often long-lasting and can affect postoperative outcome. The definition of functional recovery represents gastrointestinal function, nutritional status, physical function, and quality of life (QOL) after gastrectomy. According to research, delayed recovery is associated with a number of complications, such as prolonged hospital stay and treatment-related toxicity, leading to worse outcomes. In recent years, there has been a growing effort to analyze modifiable perioperative factors to predict or affect the postoperative recovery in gastric cancer patients [10,11,12]. The nutritional and immune status is an important factor that impacts postoperative outcomes. The tumor, dyspepsia, systemic inflammation, and catabolic stresses cause anorexia, resulting in the common occurrence of malnutrition in stomach cancer patients, which is further aggravated by surgical stress. Single parameter markers like albumin or body mass index may have limitations in their sensitivity to gauge nutritional reserves and immune status. Various immunonutritional markers have been developed and validated for predicting surgical outcomes in patients with gastric cancer [10,11,12,13,14].

The Controlling Nutritional Status (CONUT) score is a useful index with increasing cliff-edge value in clinical practice. A combination of serum albumin, total lymphocyte count, and total cholesterol is used in its calculation and it has the benefit of reproducible and objective assessment of protein reserves, immune defenses, and caloric depletion [13,14,15,16,17]. An increasing number of studies have shown that high CONUT scores are linked to high postoperative complication rates, poor recovery, and worse prognoses after gastrectomy in gastric cancer patients. Moreover, the assessment of serial CONUT during the perioperative period offered additional prognostic information that may be more informative than a single baseline assessment, since the latter may be more reflective of the effect of surgical stress and the patients’ capacity for nutritional and immunological recovery [18,19,20,21]. In this way, immunonutritional monitoring will help to estimate perioperative risk and enable individualized management. In addition, there has been a growing interest in gastric cancer and gastrectomy sex differences. Gastric cancer is more common in men, according to epidemiological evidence [10,11,12]. Recent evidence has suggested that the biological sex differences due to sex hormones, body composition, inflammatory response, and immunological function could theoretically impact gastric tumor behavior, treatment tolerability, and postoperative recovery [10,11,12]. Various cohort studies have reported sex differences in complications, functional outcomes and survival after gastrectomy with the underlying mechanisms largely unknown [10,11,12]. Despite this growing recognition, there continues to be a lack of prospective data on sex differences in postoperative nutritional and functional recovery after gastrectomy. Based on previous studies, there is some evidence on sex-based differences in postoperative nutritional and functional parameters after gastric cancer surgery. Female patients’ physical function recovery tends to be significantly quicker than male patients’ recovery in the first three months after gastrectomy. Nevertheless, a significant constraint of these preceding retrospective analyses lies in the employment of non-validated proxies for nutritional status (using body weight change), which may render the outcomes less reliable [13,14,15].

Therefore, the present study was designed as a prospective observational cohort analysis to investigate sex-related differences in nutritional status and functional recovery following subtotal gastrectomy for gastric cancer. By integrating serial CONUT assessment with clinically relevant functional outcomes, this study aims to determine whether sex represents an independent modifier of postoperative recovery trajectories and to provide evidence supporting a more personalized, sex-aware approach to perioperative care in gastric cancer surgery.

## 2. Materials and Methods

### 2.1. Study Design and Setting

This study was designed as a prospective, observational, single-center cohort investigation conducted at the General Surgery Clinic No. 1, County Emergency Clinical Hospital of Târgu Mureș and affiliated with the George Emil Palade University of Medicine, Pharmacy, Science, and Technology of Târgu Mureș, Romania. Consecutive patients undergoing curative-intent gastrectomy for gastric cancer were enrolled over the predefined study period. The prospective design allowed for standardized perioperative data collection and longitudinal assessment of nutritional and functional outcomes.

### 2.2. Ethical Approval and Informed Consent

The study protocol was reviewed and approved by the Institutional Ethics Committee of the County Emergency Clinical Hospital of Târgu Mureș (approval code 30104/7 October 2021). All procedures were conducted in accordance with the ethical standards of the Declaration of Helsinki and its later amendments. Written informed consent was obtained from all participants prior to enrollment.

### 2.3. Patient Selection

Adult patients (≥18 years) with histologically confirmed gastric adenocarcinoma who underwent elective curative-intent gastrectomy were eligible for inclusion. Both distal and total gastrectomies were considered, provided that R0 resection was achieved.

Exclusion criteria included:

- Metastatic disease at diagnosis or intraoperative evidence of unresectable disease;
- Palliative surgical procedures;
- Previous gastrectomy or prior surgery significantly altering gastrointestinal continuity;
- Severe hepatic or renal dysfunction;
- Active infection, chronic inflammatory, or autoimmune disease;
- Major psychiatric disorders impairing consent or follow-up;
- Incomplete laboratory data precluding calculation of the Controlling Nutritional Status (CONUT) score at predefined timepoints.

### 2.4. Surgical Procedure and Perioperative Management

All surgical procedures were performed by surgeons experienced in gastric cancer surgery following contemporary oncological standards. The extent of gastrectomy (distal or total) and reconstruction technique were selected intraoperatively based on tumor location, resection margins, and the feasibility of a tension-free anastomosis [4,6].

Lymphadenectomy was performed as D1+ or D2 in accordance with international and Japanese Gastric Cancer Association guidelines. Perioperative management followed standardized institutional protocols incorporating enhanced recovery principles, including early mobilization, optimized pain control, and early initiation of enteral nutrition when clinically feasible. Nutritional support was provided according to standard institutional clinical practice. However, no predefined nutritional intervention protocol based specifically on CONUT score stratification was applied during the study period. Consequently, the present investigation was designed to evaluate the prognostic value of perioperative immunonutritional status rather than the effectiveness of targeted nutritional interventions [10,16].

Neoadjuvant chemotherapy was administered selectively to patients with locally advanced disease based on multidisciplinary tumor board recommendations and international guideline criteria.

### 2.5. Nutritional and Immunological Assessment

Nutritional and immune status were evaluated using the Controlling Nutritional Status (CONUT) score, a validated composite immunonutritional index integrating serum albumin concentration, total peripheral lymphocyte count, and total cholesterol level.

Blood samples were collected at three predefined timepoints:

- T0 (preoperative): Within seven days before surgery.
- T1 (early postoperative): At postoperative day 7 ± 2 or at hospital discharge, whichever occurred first. T1 assessment was intentionally scheduled at postoperative day 7 (±2 days) or at hospital discharge because this timepoint corresponds to the early postoperative recovery phase, during which the acute metabolic and inflammatory response to surgery remains clinically relevant while immediate perioperative fluid shifts have largely stabilized. Earlier postoperative measurements (e.g., postoperative days 1–3) were not selected because they may be substantially influenced by transient hemodynamic changes, perioperative fluid administration, and acute surgical stress. The selected timepoint is consistent with previous studies evaluating perioperative immunonutritional changes following major gastrointestinal surgery.
- T3 (follow-up): Three months after surgery during routine outpatient evaluation.

Laboratory analyses were performed in the hospital’s accredited laboratory using standardized methods. The CONUT score was calculated according to established thresholds, yielding a total score ranging from 0 to 12. Nutritional status was categorized as normal (0–1), mild malnutrition (2–4), moderate malnutrition (5–8), or severe malnutrition (9–12). The detailed CONUT scoring system used in the present study is provided in Supplementary Table S1.

### 2.6. Functional Recovery and Clinical Outcomes

Functional recovery was assessed using clinically relevant postoperative endpoints, including:

- Time to restoration of gastrointestinal function;
- Length of postoperative hospital stay;
- Postoperative complications occurring within 30 days, classified according to the Clavien–Dindo grading system;
- Thirty-day readmission rate;
- Thirty-day postoperative mortality.

Baseline demographic and clinical variables, including age, sex, body mass index, comorbidities, American Society of Anesthesiologists (ASA) score, tumor location, and surgical details, were prospectively recorded. For multivariable analysis, delayed functional recovery was defined as a composite binary outcome consisting of at least one of the following: postoperative hospital stay exceeding the cohort median value (>10 days), delayed restoration of gastrointestinal function (time to first flatus > 4 days), or occurrence of major postoperative complications (Clavien–Dindo grade III or higher). Patients fulfilling any of these criteria were classified as having delayed functional recovery.

### 2.7. Statistical Analysis

Statistical analyses were performed using EasyMedStat (EasyMedStat SAS, Paris, France). Data distribution was assessed using the Shapiro–Wilk test. Continuous variables were expressed as mean ± standard deviation for normally distributed data or median with interquartile range for non-normally distributed data. Categorical variables were presented as frequencies and percentages.

Comparisons between male and female patients were conducted using the independent-samples *t*-test or the Mann–Whitney U test for continuous variables and the χ^2^ test or Fisher’s exact test for categorical variables, as appropriate. Longitudinal changes in immunonutritional parameters (serum albumin, lymphocyte count, total cholesterol, and CONUT score) were evaluated using repeated-measures mixed-effects models. Time (T0, T1, and T3) was treated as a within-subject factor, while sex was included as a between-subject factor. Interaction effects between sex and time were assessed to determine whether postoperative recovery trajectories differed according to biological sex. Patient identification number was included as a random effect to account for within-subject correlations arising from repeated measurements.

Because mixed-effects models are robust to unbalanced longitudinal data, patients with incomplete follow-up assessments were retained whenever at least one postoperative measurement was available. Missing observations were not imputed. The primary analyses were adjusted for clinically relevant covariates, including age, ASA class, pathological stage, reconstruction type, and neoadjuvant chemotherapy, when appropriate. Multivariable logistic regression models were constructed to identify independent predictors of delayed functional recovery. Variables were selected based on their clinical relevance and results of univariable analyses. Variables demonstrating a univariable association with *p* < 0.10, together with clinically important covariates identified from the previous literature (age, sex, ASA class, nutritional status, tumor stage, reconstruction type, and neoadjuvant chemotherapy), were entered into the final multivariable model. Adjusted odds ratios (ORs) with corresponding 95% confidence intervals (CIs) were calculated. All statistical tests were two-tailed, and a *p* value < 0.05 was considered statistically significant.

Artificial intelligence (AI)-assisted technology was used exclusively during the final manuscript preparation stage. Specifically, ChatGPT-5 (OpenAI, San Francisco, CA, USA) was employed for English language editing, grammatical correction, sentence restructuring, and readability improvement. No AI tool was used for study design, patient recruitment, data collection, statistical analysis, interpretation of results, generation of scientific conclusions, or preparation of original scientific content. All scientific concepts, analyses, and interpretations were independently developed, verified, and approved by the authors.

## 3. Results

**Figure 1:**
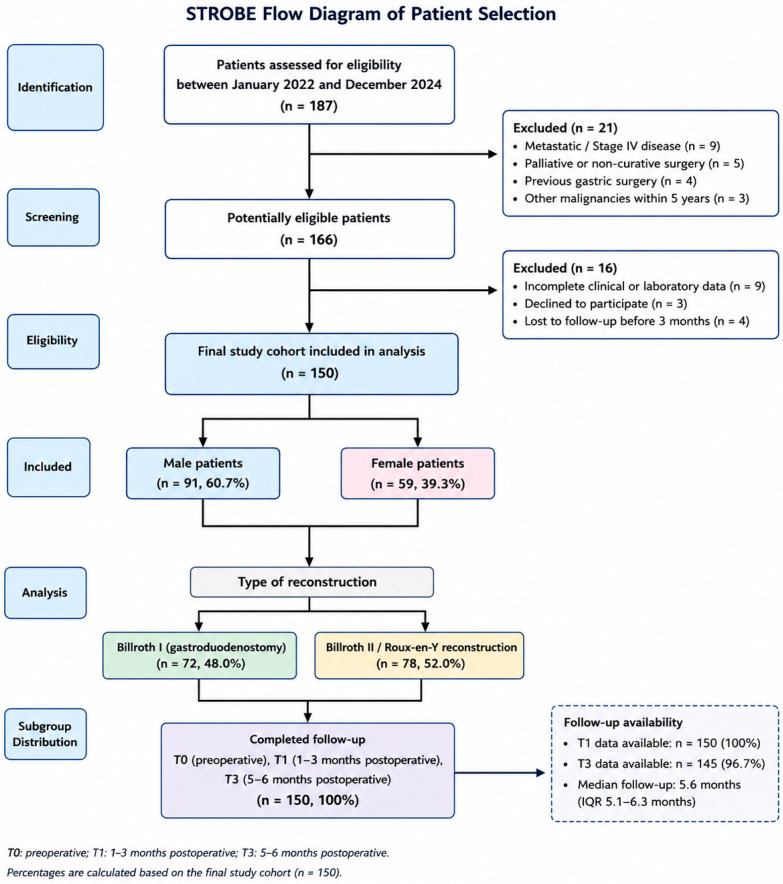
STROBE flow diagram illustrating patient selection, exclusion criteria, sex distribution, reconstruction type, and final inclusion of patients in the prospective cohort analysis.

A total of 187 patients undergoing evaluation for curative-intent gastrectomy for gastric cancer were initially assessed for eligibility during the study period. After exclusion of patients with metastatic disease, palliative procedures, previous gastric surgery, incomplete laboratory data, or loss to follow-up, 150 patients were included in the final prospective cohort analysis. The study population consisted of 91 male patients (60.7%) and 59 female patients (39.3%). Billroth I reconstruction was performed in 72 patients (48.0%), whereas 78 patients (52.0%) underwent Billroth II or Roux-en-Y reconstruction (Figure 1).

### 3.1. Baseline Clinicopathological Characteristics

The average age of the group was 62.4 ± 11.8 years, with no statistically significant age difference between male and female patients (63.1 ± 11.5 vs. 61.3 ± 12.2 years, *p* = 0.32). Likewise, body mass index was not significantly different by sex (25.0 ± 3.7 vs. 24.5 ± 4.1 kg/m^2^, *p* = 0.41). A significant majority of the patients were classified as ASA I–II (65.3%). Quite a few patients, 34.7%, presented ASA III status. No sex-related differences were noted (*p* = 0.84). Cardiovascular comorbidities were present in 55.3% of enrolled patients, while diabetes mellitus was present in 27.3% of enrolled patients with a balanced male and female distribution. With regard to oncological features, distal/antral tumors were the most frequent tumor site (72.7%), whilst stage I–II disease was found in 58.7% of patients and stage III in 41.3%. Male and female patients did not significantly differ regarding tumor location or pathological stage (*p* > 0.05 for all comparisons). In 30.7% of cases, neoadjuvant chemotherapy was used, with no significant gender difference. Immunonutritional baseline parameters were shown. Similarly, the distribution of CONUT classification at T0 did not differ according to sex (*p* = 0.88) [Table 1]. Five patients were unavailable for complete T3 assessment because of mortality or loss to follow-up; therefore, longitudinal analyses at the 3-month follow-up timepoint were performed in 145 patients.

### 3.2. Perioperative and Short-Term Postoperative Outcomes

The mean operative time for the entire cohort was 201.4 ± 34.8 min, while mean estimated blood loss was 312.6 ± 96.3 mL. No significant sex-related differences were identified regarding operative duration or intraoperative blood loss.

Postoperative recovery parameters were also comparable between sexes. The mean time to first flatus was 3.8 ± 1.2 days, and oral diet was resumed after a mean interval of 5.9 ± 1.7 days. The mean postoperative hospital stay was 10.8 ± 3.9 days, without significant variation between male and female patients (*p* = 0.48).

Overall postoperative complications occurred in 31.3% of patients. Most complications were classified as Clavien–Dindo grade I–II (22.0%), while major complications (grade III–IV) occurred in 9.3% of cases. The overall complication profile did not significantly differ according to sex (*p* = 0.77). Anastomotic leakage occurred in nine patients (6.0%). Most leaks were diagnosed during the first postoperative week based on clinical deterioration and radiological findings. Management strategies included image-guided percutaneous drainage, endoscopic treatment when appropriate, and surgical reintervention in selected cases. Anastomotic leakage represented one of the most clinically significant postoperative complications and was associated with prolonged hospitalization and delayed recovery. Surgical site infection occurred in 9.3% of patients, postoperative ileus in 7.3%, and pulmonary complications in 8.7%, with no statistically significant sex-related differences observed.

The 30-day readmission and reoperation rates were 8.0% and 6.7%, respectively, while overall 90-day mortality was 2.7%. These outcomes were similarly distributed between male and female patients (Table 2).

### 3.3. Longitudinal Evolution of Immunonutritional Parameters

Significant temporal variation was observed for all evaluated nutritional and immunological markers. Serum albumin levels demonstrated a marked postoperative decline from 3.62 ± 0.54 g/dL at baseline (T0) to 3.18 ± 0.47 g/dL during the early postoperative period (T1), followed by partial recovery at the 3-month follow-up evaluation (T3: 3.71 ± 0.43 g/dL; *p*-time < 0.001). Similar temporal patterns were identified for lymphocyte count and total cholesterol level, both of which significantly deteriorated immediately after surgery and subsequently improved during follow-up (*p*-time < 0.001 for all comparisons).

**Figure 2:**
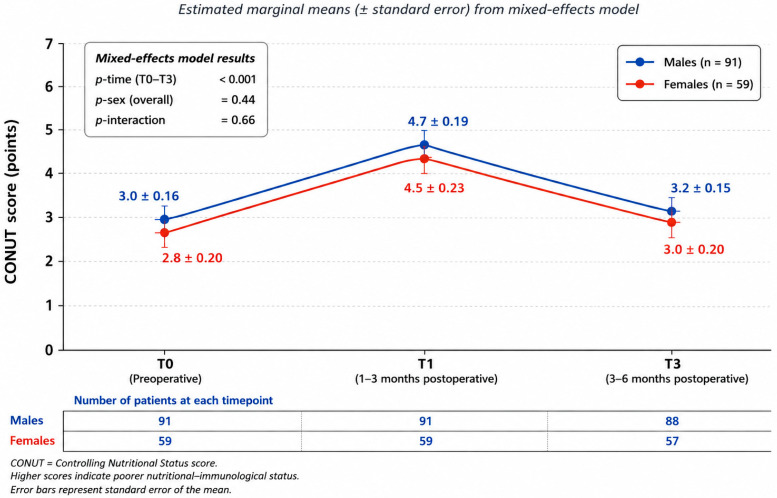
Longitudinal evolution of the CONUT score according to sex across the predefined perioperative timepoints (T0, preoperative; T1, early postoperative; T3, 3-month follow-up). Both groups demonstrated significant postoperative deterioration followed by partial recovery during follow-up, without significant sex-related interaction effects. The reduced number of patients available at T3 (*n* = 145) reflects loss to follow-up and mortality occurring during the study period, as detailed in the STROBE flow diagram (Figure 1).

The CONUT score showed significant postoperative worsening, increasing from 2.9 ± 1.6 at T0 to 4.6 ± 1.9 at T1, followed by improvement at T3 (3.1 ± 1.5; *p*-time < 0.001). However, despite postoperative recovery, immunonutritional parameters did not completely return to preoperative values in a subset of patients. No statistically significant overall sex effect was identified for albumin, lymphocyte count, cholesterol level, or CONUT score (*p*-sex > 0.05). Furthermore, repeated-measures mixed-effects analyses demonstrated no significant interaction between sex and longitudinal evolution of immunonutritional status (*p*-interaction > 0.05), indicating comparable postoperative nutritional recovery trajectories in male and female patients [Table 3, Figure 2].

### 3.4. Functional and Mid-Term Recovery Outcomes

Mean postoperative weight loss at the 3-month follow-up evaluation was 8.6 ± 4.1%. At follow-up endoscopic evaluation, LA esophagitis was absent in 58.0% of patients, while grade A–B esophagitis was identified in 32.0% and grade C–D esophagitis in 10.0% of cases. Bile reflux gastritis was absent in 52.7% of patients, whereas mild and moderate–severe forms were identified in 27.3% and 20.0% of cases, respectively. No statistically significant sex-related differences were observed regarding reflux-related complications.

Dumping syndrome, defined as a Sigstad score > 7, was identified in 24.0% of patients, while proton pump inhibitor use remained necessary in 38.7% of patients at 3 months and 29.3% at 6 months postoperatively. These functional outcomes were similarly distributed between male and female patients (Table 4).

### 3.5. Independent Predictors of Delayed Functional Recovery

**Figure 3:**
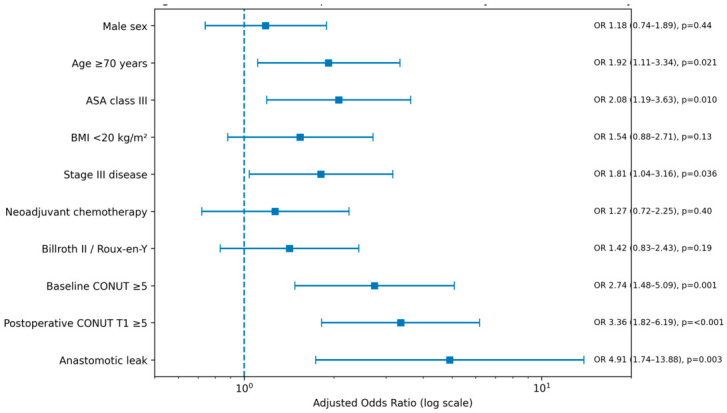
Forest plot illustrating independent predictors of delayed functional recovery after gastrectomy based on multivariable logistic regression analysis. Elevated postoperative CONUT score (T1 ≥ 5), baseline immunonutritional impairment, ASA class III status, advanced age, stage III disease, and anastomotic leakage were independently associated with adverse postoperative recovery outcomes. Biological sex, reconstruction type, body mass index, and neoadjuvant chemotherapy were not identified as independent predictors after multivariable adjustment. Odds ratios are presented on a logarithmic scale with corresponding 95% confidence intervals.

Advanced age (≥70 years), ASA class III, stage III disease, elevated baseline CONUT score (≥5), and postoperative deterioration of immunonutritional status (CONUT T1 ≥ 5) emerged as independent predictors of delayed functional recovery. Among the evaluated variables, postoperative CONUT deterioration demonstrated one of the strongest independent associations with impaired postoperative recovery (OR 3.36, 95% CI 1.82–6.19, *p* < 0.001). Anastomotic leakage was also strongly associated with delayed recovery (OR 4.91, 95% CI 1.74–13.88, *p* = 0.003). The strong association between anastomotic leakage and delayed recovery is clinically expected, given its impact on nutritional intake, systemic inflammatory response, prolonged hospitalization, and the need for additional therapeutic interventions. These findings further emphasize the importance of early recognition and aggressive management of postoperative leaks within modern gastric cancer recovery pathways. After adjustment for relevant demographic, oncological, and perioperative covariates, sex was not independently associated with delayed postoperative recovery (OR 1.18, 95% CI 0.74–1.89, *p* = 0.44). These findings suggest that perioperative immunonutritional status and postoperative complications exert a greater influence on recovery trajectories than biological sex alone (Table 5, Figure 3).

An additional exploratory subgroup analysis was performed according to both biological sex and preoperative CONUT category. In both male and female patients, increasing baseline immunonutritional impairment was associated with progressively higher rates of postoperative complications, prolonged hospital stays, delayed gastrointestinal recovery, and delayed functional recovery. Patients presenting with moderate-to-severe preoperative CONUT scores demonstrated the least favorable postoperative outcomes regardless of sex. Detailed outcome comparisons according to sex and baseline CONUT category are presented in Supplementary Table S2.

## 4. Discussion

The present prospective cohort study examined the sex-related differences in nutritional status, immunological recovery, and postoperative functional outcomes after curative-intent gastrectomy for gastric cancer. This study evaluated postoperative recovery trajectories in a modern gastric cancer population through serial perioperative evaluation of the Controlling Nutritional Status (CONUT) score with other clinically relevant recovery parameters. We can summarize the principal findings of the current study as follows:

First, the postoperative deterioration in immunonutritional status occurred to a significant extent in both males and females. Second, the longitudinal recovery patterns during the follow-up investigation were comparable in males and females. Third, biological sex was not independently associated with delayed functional recovery after adjustment for the relevant clinicopathological parameters. Last, perioperative immunonutritional impairment, particularly increased postoperative CONUT score, was among the most potent independent predictors of delayed recovery after surgery for gastric cancer. Notwithstanding the significant advancement in surgical technique, perioperative care, and enhanced recovery after surgery protocols [4,5,6,7,8], metabolic and physiological stress in gastric cancer surgery is considerable. Gastrectomy leads to significant alterations in gastrointestinal continuity, digestive functionality, nutrient uptake, and overall homeostasis of systemic inflammation, creating a complex postoperative picture, with transient immunosuppression, protein–energy deficit, and nutritional depletion [8,21,22].

In the present cohort, we noticed that serum albumin and lymphocyte and total cholesterol counts showed significant early postoperative deterioration followed by only partial recovery at the 3-month follow-up evaluation. According to the findings, postoperative catabolic response following gastrectomy occurs not only in the immediate perioperative period but it also takes place for a relatively long time after surgery. This evidence is consistent with earlier studies [14,15,16,17,21,22,23]. The increase in CONUT scores we observed after surgery is also in accordance with the concept of a clinically relevant degradation of immunonutritional status after gastrectomy. We noticed that, during follow-up, recovery only occurs partway among patients, and not every patient returns to baseline nutritional parameters. According to this study, gastrectomy is indeed a surgical procedure with metabolic effects that persist long after surgery. The findings of our research are consistent with established studies evaluating the prognostic effect of perioperative CONUT dynamics in gastric surgery [12,13,14,15,16,17,18,19,20]. Numerous studies have demonstrated that a higher CONUT score relates to the increase in postoperative complications, longer hospital stays, reduced tolerance to administration of chemotherapy agents, and inferior oncological outcomes [13,14,15,16,17,18,19,20]. As such, our findings further support the ever-growing body of literature in favor of serial immunonutritional monitoring being a requirement for perioperative risk stratification for gastric cancer.

To prospectively assess the impact of biological sex on postoperative immunonutritional recovery was one of the goals of current study. According to epidemiological studies, there are important sex differences in the incidence of gastric cancer. The disease shows a clearer male predilection as per worldwide descriptions. Furthermore, sex hormones, inflammatory processes, immunomodulatory mechanisms, body composition, and vulnerability to sarcopenia may play a role in sex-specific cancer and postoperative outcomes [9,10,11]. Insufficient evidence exists in the literature on the impact of sex regarding postoperative infusional immunonutrition. The study shows baseline clinicopathological and immunonutritional characteristics were similar among males and females. Also, there was no substantial sex-related difference concerning operative variables, complications, gastrointestinal recovery, reflux-related outcomes, or CONUT evolution. Repeated-measures analyses did not show interaction of sex with recovery of nutritional status after surgery. This suggests that the effects of gastrectomy on immunonutritional status can be more important than biological heterogeneity due to sex in the early and intermediate postoperative period. The absence of significant sex-related differences should also be interpreted in relation to the nutritional assessment method used. Although the CONUT score is a validated and clinically accessible marker of global immunonutritional status, it may not fully capture more subtle sex-specific differences in postoperative recovery related to body composition, skeletal muscle mass, sarcopenia, hormonal status, or patient-reported physical function. In the present cohort, CONUT demonstrated a clear postoperative deterioration followed by partial recovery, confirming its sensitivity to perioperative immunonutritional changes. However, the similar temporal pattern observed in both sexes suggests that the metabolic impact of gastrectomy may outweigh sex-specific biological variability during the early postoperative recovery period. The findings are partly consistent with previous large cohort studies, which determined that sex made a limited independent contribution to short-term postoperative outcomes after gastric cancer surgery [9,10]. According to Arakawa et al., although there were clinicopathological differences between males and females, postoperative morbidity and recovery were not significantly altered when adjusting for confounders [11]. National analyses have suggested that sex may not have independent predictive association with postoperative complications after adjusting for tumor stage, comorbidities, nutritional status, and operative complexity. Other previous studies suggested female sex to be a risk factor for postoperative complications after gastrectomy [11]. However, these differences may have originated from the heterogeneity of study populations, retrospective nature, differences in protocols for perioperative management and lack of standard immunonutritional assessment tools.

An important finding of the present study is the strong independent association between impaired perioperative immunonutritional status and delayed functional recovery. Higher baseline CONUT score and increased CONUT after operation were independent predictors of poor recovery after multivariable adjustment. Postoperative CONUT deterioration in particular was associated with some of the highest odds ratios among the different predictors evaluated, even exceeding the effect of biological sex or reconstruction type. Therefore, clinical determinants of recovery after surgery may be economic status, nutrition, or immunity rather than age or gender. According to the pathophysiological hypothesis, the predictive value of CONUT is due to the multifactorial interplay of protein depletion, impaired immune competence, and reduced metabolic adaptive potential. Hypoalbuminemia can have a negative effect due to its role in hampering wound healing, susceptibility to infection, and prolongation of inflammatory response while lymphopenia indicates perioperative immunosuppression and reduced cellular immune defense [12,13,14,15,16,17,18,19,20]. Assessment of caloric depletion is a bit more difficult; impairment of the lipid metabolism could have also affected postoperative tissue repair and functional recovery. The CONUT score may provide a more complete evaluation of surgical risk than individual laboratory variables. Neoadjuvant chemotherapy may also influence perioperative nutritional status through treatment-related metabolic alterations, appetite reduction, and systemic inflammatory effects. Although neoadjuvant therapy was not independently associated with delayed functional recovery in the present cohort, the study was not specifically powered to investigate its isolated impact on longitudinal immunonutritional recovery. Future studies should further explore the interaction between preoperative systemic treatment and postoperative nutritional trajectories. The current findings also have significant clinical implications for managing perioperative procedures. These findings may have direct implications for enhanced recovery after surgery (ERAS) protocols in gastric cancer surgery. The strong association between postoperative immunonutritional deterioration and delayed functional recovery suggests that serial CONUT assessment could serve as a practical tool for early risk stratification during the postoperative period. Patients presenting with elevated postoperative CONUT scores (≥5) may benefit from intensified nutritional surveillance, early dietitian involvement, individualized dietary counseling, and targeted immunonutritional supplementation. Incorporating dynamic immunonutritional monitoring into ERAS pathways may facilitate earlier identification of vulnerable patients and support more personalized postoperative recovery strategies. From a practical perspective, serial CONUT monitoring could be readily integrated into existing ERAS protocols because all three components of the score (serum albumin concentration, total lymphocyte count, and total cholesterol level) are routinely available laboratory parameters. Preoperative CONUT assessment may assist in identifying patients requiring nutritional optimization before surgery, while postoperative reassessment could help detect individuals experiencing significant immunonutritional deterioration. Such patients may benefit from early dietitian referral, intensified nutritional support, immunonutritional supplementation, and closer postoperative surveillance. Consequently, serial CONUT assessment represents a simple, inexpensive, and reproducible strategy for supporting personalized recovery pathways following gastrectomy.

The assessment of immunonutritional status using the CONUT score offers several practical advantages, including objectivity, simplicity, reproducibility, and reliance on routinely available laboratory parameters. Consequently, it represents an accessible tool for perioperative risk stratification in everyday clinical practice. Nevertheless, CONUT does not directly evaluate important dimensions of nutritional status such as skeletal muscle mass, sarcopenia, body composition, frailty, or patient-reported functional capacity. Therefore, it should be interpreted as a complementary marker rather than a comprehensive assessment of nutritional risk. In the present cohort, impaired perioperative CONUT scores were associated with less favorable postoperative recovery trajectories, whereas biological sex was not identified as an independent predictor of postoperative outcomes. These findings suggest that perioperative immunonutritional status may have greater clinical relevance than sex alone for predicting short- and intermediate-term recovery following gastrectomy for gastric cancer. The additional subgroup analyses demonstrated that higher baseline CONUT scores were associated with progressively longer postoperative hospitalization and less favorable recovery profiles. Conversely, no clinically meaningful differences in postoperative outcomes were observed between male and female patients after adjustment for perioperative factors, suggesting that immunonutritional status exerts a greater influence on postoperative recovery than biological sex alone.

Modern surgical practices for gastric cancer have evolved towards adopting personalized perioperative strategies aimed at optimizing the postoperative recovery while reducing treatment related functional deterioration [4,7,8,21]. According to our findings, systematic perioperative immunonutritional assessment might help facilitate an early timely detection of patients at risk of delayed recovery regardless of sex. In the same manner, continual monitoring of CONUT can provide an easy and low-cost addition to postoperative monitoring.

Also, the lack of sex-specific differences in our cohort suggests that optimizing modifiable physiological rather than exclusively demographic factors should be the focus of any perioperative optimizing strategy. The impact of early nutrition, perioperative immunonutrition, enhanced recovery pathways and aggressive prevention of postoperative complications is likely to be much greater than sex-specific biological variability. The extent of gastric resection may also influence postoperative nutritional adaptation and long-term recovery. Although both distal and total gastrectomy patients were included in the present cohort, the study was not specifically designed or powered to evaluate differences according to resection extent. Future studies with larger patient populations should investigate whether total gastrectomy is associated with distinct immunonutritional recovery trajectories compared with distal gastrectomy.

Future research that considers relationships between body composition, sarcopenia, inflammatory cytokines, and hormonal assessment may further elucidate whether more subtle gender-related biological mechanisms influence long-term recovery, and/or oncological outcomes after gastrectomy. In addition, larger studies may be warranted to explore potential interaction effects between biological sex and other clinicopathological variables, including age, tumor stage, reconstruction type, and neoadjuvant treatment. Such analyses may help identify more subtle sex-specific determinants of postoperative recovery that could not be fully evaluated within the present cohort.

There are various limitations in the current study. To begin with, the present study was a single-center observational study which may affect external and internal validity to other populations and health systems. In addition, while standardized data collection and the prospective design were an advantage, the sample size may have limited the detection of smaller sex related differences. Furthermore, although all consecutive eligible patients treated during the predefined study period were prospectively enrolled, no formal a priori sample size calculation specifically targeting sex-related differences was performed. Consequently, the study may have been underpowered to detect subtle sex-specific variations in postoperative nutritional and functional recovery, particularly given the smaller number of female patients included in the cohort. Therefore, the absence of statistically significant sex-related associations should be interpreted with caution and does not exclude the possibility of smaller clinically relevant effects. Third, follow-up was limited to the early and intermediate postoperative period and therefore did not permit assessment of long-term nutritional adaptation, late functional recovery, chronic quality-of-life outcomes, or long-term oncological results. Extended follow-up studies are needed to determine whether the observed immunonutritional recovery trajectories persist over time and whether they influence long-term clinical outcomes after gastrectomy. Ultimately, inflammatory biomarkers, such as C-reactive protein, interleukin-6, or cytokine profiling, were not integrated in a systematic way into the perioperative analysis. Research utilizing metanalyses to compare Billroth I, Billroth II, and Roux-en-Y reconstructions have shown that, while there was a significant mix observed in their impact on reflux symptoms, nutritional recovery, and the postoperative quality of life with each method, none of the metanalyses seemed to demonstrate the superiority of one reconstructive method over the other [24,25,26,27]. Nevertheless, the present study had various strengths such as prospective design, uniform perioperative follow-up with longitudinal immunonutritional assessment, and incorporation of functionally meaningful clinical outcomes. To the best of our knowledge, this is one of the rare prospective studies directly investigating sex-related differences in postoperative immunonutritional recovery using serial CONUT evaluation among patients of gastric cancer undergoing gastrectomy.

A more personalized postoperative management approach could be achieved through the methodical integration of a dynamic immunonutritional monitoring instrument. This, in turn, enhances recovery protocols and supports an improved functional recovery after gastrectomy.

## 5. Conclusions

Patients undergoing subtotal distal gastrectomy for gastric cancer experienced a significant deterioration in immunonutritional status during the early postoperative period, with only partial recovery reported at intermediate follow-up. The current prospective cohort of male and female patients exhibited similar longitudinal trajectories of nutritional parameters, postoperative functional outcomes, and complication profiles. Despite biological differences between the sexes being increasingly acknowledged in gastric cancer, biological sex per se was not associated with delayed postoperative recovery when adjusted for relevant clinicopathological and perioperative factors.

On the other hand, suboptimal perioperative immunonutritional condition, especially with a raised postoperative CONUT score, was found to be a significant independent risk factor for negative functional recovery following gastrectomy. Moreover, this suggests that dynamic changes in nutritional and immune reserve are important in determining postoperative recovery trajectories and is excellent justification for serial immunonutritional assessment in standard perioperative management of patients with gastric cancer. Regular monitoring of CONUT levels during surgery can help assess risk and provide better treatment.

## Figures and Tables

**Table 1:** Baseline clinicopathological characteristics of the study population stratified by sex (*n* = 150).

Variable	Total (*n* = 150)	Males (*n* = 91)	Females (*n* = 59)	*p*-Value
Age, years	62.4 ± 11.8	63.1 ± 11.5	61.3 ± 12.2	0.32 †
Body mass index, kg/m^2^	24.8 ± 3.9	25.0 ± 3.7	24.5 ± 4.1	0.41 †
ASA class				0.84 ‡
ASA I–II, *n* (%)	98 (65.3%)	60 (65.9%)	38 (64.4%)	
ASA III, *n* (%)	52 (34.7%)	31 (34.1%)	21 (35.6%)	
Comorbidities, *n* (%)				
Cardiovascular disease	83 (55.3%)	52 (57.1%)	31 (52.5%)	0.59 ‡
Diabetes mellitus	41 (27.3%)	26 (28.6%)	15 (25.4%)	0.68 ‡
Tumor location, *n* (%)				0.74 ‡
Antrum/distal stomach	109 (72.7%)	67 (73.6%)	42 (71.2%)	
Pathological stage, *n* (%)				0.81 ‡
Stage I–II	88 (58.7%)	54 (59.3%)	34 (57.6%)	
Stage III	62 (41.3%)	37 (40.7%)	25 (42.4%)	
Neoadjuvant chemotherapy, *n* (%)	46 (30.7%)	29 (31.9%)	17 (28.8%)	0.69 ‡
Type of reconstruction, *n* (%)				0.91 ‡
Billroth I	72 (48.0%)	44 (48.4%)	28 (47.5%)	
Billroth II/Roux-en-Y	78 (52.0%)	47 (51.6%)	31 (52.5%)	
Albumin T0, g/dL	3.62 ± 0.54	3.60 ± 0.52	3.65 ± 0.56	0.47 †
Lymphocytes T0, ×10^9^/L	1.78 ± 0.46	1.75 ± 0.44	1.82 ± 0.48	0.36 †
Total cholesterol T0, mg/dL	168.3 ± 34.7	166.9 ± 33.9	170.4 ± 36.1	0.52 †
CONUT score T0	2.9 ± 1.6	3.0 ± 1.5	2.8 ± 1.7	0.44 †
CONUT category T0, *n* (%)				0.88 ‡
Normal (0–1)	49 (32.7%)	29 (31.9%)	20 (33.9%)	
Mild (2–4)	71 (47.3%)	44 (48.4%)	27 (45.8%)	
Moderate–Severe (≥5)	30 (20.0%)	18 (19.8%)	12 (20.3%)	

† Independent-samples *t*-test. ‡ χ^2^ test or Fisher’s exact test, as appropriate.

**Table 2:** Perioperative and short-term postoperative outcomes stratified by sex (*n* = 150).

Variable	Total (*n* = 150)	Males (*n* = 91)	Females (*n* = 59)	*p*-Value
Operative time, min	201.4 ± 34.8	203.1 ± 35.2	198.8 ± 34.1	0.47 †
Estimated blood loss, mL	312.6 ± 96.3	318.5 ± 99.1	303.4 ± 91.7	0.36 †
Time to first flatus, days	3.8 ± 1.2	3.9 ± 1.3	3.7 ± 1.1	0.41 †
Time to oral diet, days	5.9 ± 1.7	6.0 ± 1.8	5.8 ± 1.6	0.52 †
Length of hospital stay, days	10.8 ± 3.9	11.0 ± 4.1	10.5 ± 3.6	0.48 †
Overall postoperative complications, *n* (%)	47 (31.3%)	30 (33.0%)	17 (28.8%)	0.59 ‡
Clavien–Dindo classification, *n* (%)				0.77 ‡
Grade I–II	33 (22.0%)	21 (23.1%)	12 (20.3%)	
Grade III–IV	14 (9.3%)	9 (9.9%)	5 (8.5%)	
Grade V	2 (1.3%)	1 (1.1%)	1 (1.7%)	
Specific complications, *n* (%)				
Anastomotic leak	9 (6.0%)	6 (6.6%)	3 (5.1%)	0.71 §
Surgical site infection	14 (9.3%)	9 (9.9%)	5 (8.5%)	0.78 ‡
Postoperative ileus	11 (7.3%)	7 (7.7%)	4 (6.8%)	0.84 §
Pulmonary complications	13 (8.7%)	9 (9.9%)	4 (6.8%)	0.51 §
30-day readmission, *n* (%)	12 (8.0%)	8 (8.8%)	4 (6.8%)	0.66 §
30-day reoperation, *n* (%)	10 (6.7%)	6 (6.6%)	4 (6.8%)	0.97 §
90-day mortality, *n* (%)	4 (2.7%)	3 (3.3%)	1 (1.7%)	0.56 §

† Independent-samples *t*-test. ‡ χ^2^ test. § Fisher’s exact test.

**Table 3:** Longitudinal evolution of immunonutritional parameters according to sex.

Variable	Timepoint	Total (*n* = 150)	Males (*n* = 91)	Females (*n* = 59)	*p*-Value	*p*-Sex
Albumin, g/dL	T0	3.62 ± 0.54	3.60 ± 0.52	3.65 ± 0.56	<0.001	0.41 †
	T1	3.18 ± 0.47	3.15 ± 0.45	3.22 ± 0.49		
	T3	3.71 ± 0.43	3.68 ± 0.41	3.75 ± 0.46		
Lymphocytes, ×10^9^/L	T0	1.78 ± 0.46	1.75 ± 0.44	1.82 ± 0.48	<0.001	0.36 †
	T1	1.34 ± 0.39	1.31 ± 0.37	1.38 ± 0.42		
	T3	1.69 ± 0.41	1.66 ± 0.40	1.73 ± 0.43		
Total cholesterol, mg/dL	T0	168.3 ± 34.7	166.9 ± 33.9	170.4 ± 36.1	<0.001	0.52 †
	T1	142.7 ± 31.5	140.9 ± 30.8	145.4 ± 32.3		
	T3	161.5 ± 33.7	159.8 ± 32.9	164.1 ± 34.6		
CONUT score	T0	2.9 ± 1.6	3.0 ± 1.5	2.8 ± 1.7	<0.001	0.44 †
	T1	4.6 ± 1.9	4.7 ± 1.8	4.5 ± 2.0		
	T3	3.1 ± 1.5	3.2 ± 1.4	3.0 ± 1.6		

† Between-subject sex effect.

**Table 4:** Functional and nutritional outcomes at follow-up stratified by sex (*n* = 150).

Variable	Total (*n* = 150)	Males (*n* = 91)	Females (*n* = 59)	*p*-Value
Weight loss at 3 months, %	8.6 ± 4.1	8.9 ± 4.3	8.1 ± 3.8	0.29 †
Albumin T1, g/dL	3.18 ± 0.47	3.15 ± 0.45	3.22 ± 0.49	0.38 †
Albumin T3, g/dL	3.71 ± 0.43	3.68 ± 0.41	3.75 ± 0.46	0.34 †
Lymphocytes T1, ×10^9^/L	1.34 ± 0.39	1.31 ± 0.37	1.38 ± 0.42	0.31 †
Lymphocytes T3, ×10^9^/L	1.69 ± 0.41	1.66 ± 0.40	1.73 ± 0.43	0.37 †
Total cholesterol T1, mg/dL	142.7 ± 31.5	140.9 ± 30.8	145.4 ± 32.3	0.41 †
Total cholesterol T3, mg/dL	161.5 ± 33.7	159.8 ± 32.9	164.1 ± 34.6	0.46 †
CONUT score T1	4.6 ± 1.9	4.7 ± 1.8	4.5 ± 2.0	0.52 †
CONUT score T3	3.1 ± 1.5	3.2 ± 1.4	3.0 ± 1.6	0.47 †
LA esophagitis, *n* (%)				0.73 ‡
Absent	87 (58.0%)	52 (57.1%)	35 (59.3%)	
Grade A–B	48 (32.0%)	30 (33.0%)	18 (30.5%)	
Grade C–D	15 (10.0%)	9 (9.9%)	6 (10.2%)	
Bile reflux gastritis, *n* (%)				0.68 ‡
Absent	79 (52.7%)	47 (51.6%)	32 (54.2%)	
Mild	41 (27.3%)	26 (28.6%)	15 (25.4%)	
Moderate–Severe	30 (20.0%)	18 (19.8%)	12 (20.3%)	
Dumping syndrome (Sigstad score > 7), *n* (%)	36 (24.0%)	23 (25.3%)	13 (22.0%)	0.65 ‡
PPI use at 3 months, *n* (%)	58 (38.7%)	36 (39.6%)	22 (37.3%)	0.78 ‡
PPI use at 6 months, *n* (%)	44 (29.3%)	27 (29.7%)	17 (28.8%)	0.91 ‡

† Independent-samples *t*-test. ‡ χ^2^ test or Fisher’s exact test, as appropriate.

**Table 5:** Multivariable logistic regression analysis for predictors of delayed functional recovery after gastrectomy.

Variable	Adjusted OR	95% CI	*p* Value
Male sex	1.18	0.74–1.89	0.44
Age ≥ 70 years	1.92	1.11–3.34	0.021
ASA class III	2.08	1.19–3.63	0.010
Body mass index < 20 kg/m^2^	1.54	0.88–2.71	0.13
Stage III disease	1.81	1.04–3.16	0.036
Neoadjuvant chemotherapy	1.27	0.72–2.25	0.40
Billroth II/Roux-en-Y reconstruction	1.42	0.83–2.43	0.19
Baseline CONUT score ≥ 5	2.74	1.48–5.09	0.001
Postoperative CONUT score T1 ≥ 5	3.36	1.82–6.19	<0.001
Anastomotic leak	4.91	1.74–13.88	0.003

## Data Availability

The data that support the findings of this study are available from the main author, C.C., upon request via catalin.cosma@umfst.ro, within the limits of the ethical considerations of the hospital commission.

## Supplementary Materials

The following supporting information can be downloaded at: https://www.mdpi.com/article/10.3390/jcm15124558/s1, Supplementary Table S1. Components and scoring criteria of the Controlling Nutritional Status (CONUT) score. Interpretation of Total CONUT Score. Supplementary Table S2. Baseline preoperative CONUT category distribution and postoperative recovery according to sex.

## Abbreviations

ASA	American Society of Anesthesiologists
BMI	Body Mass Index
CI	Confidence Interval
CONUT	Controlling Nutritional Status
ERAS	Enhanced Recovery After Surgery
JGCA	Japanese Gastric Cancer Association
LOS	Length of Stay
OR	Odds Ratio
PPI	Proton Pump Inhibitor
R0	Microscopically Margin-Negative Resection
T0	Preoperative Timepoint
T1	Early Postoperative Timepoint
T3	Three-Month Follow-Up Timepoint
