# Supplementary material for: Perioperative Immunonutritional Status and Functional Recovery After Gastrectomy for Gastric Cancer: A Prospective Cohort Study of Sex-Related Differences
Source: J Clin Med. 2026 Jun 12;15(12):4558. doi: 10.3390/jcm15124558 (PMC13302580; doi:10.3390/jcm15124558)
Supplement: Supplementary file 1 [file jcm-15-04558-s001.zip › Supplementary_Table_S1.pdf]

Supplementary Table S1

Components and scoring criteria of the Controlling Nutritional Status (CONUT) score

Parameter	Value	Score
**Serum albumin (g/dL)**	≥3.5	0
	3.0–3.49	2
	2.5–2.99	4
	<2.5	6
**Total lymphocyte count (/mm³)**	≥1600	0
	1200–1599	1
	800–1199	2
	<800	3
**Total cholesterol (mg/dL)**	≥180	0
	140–179	1
	100–139	2
	<100	3

Interpretation of Total CONUT Score

Total Score	Nutritional Status
**0–1**	Normal
**2–4**	Mild malnutrition
**5–8**	Moderate malnutrition
**9–12**	Severe malnutrition
