# Supplementary material for: Perioperative Immunonutritional Status and Functional Recovery After Gastrectomy for Gastric Cancer: A Prospective Cohort Study of Sex-Related Differences
Source: J Clin Med. 2026 Jun 12;15(12):4558. doi: 10.3390/jcm15124558 (PMC13302580; doi:10.3390/jcm15124558)
Supplement: Supplementary file 1 [file jcm-15-04558-s001.zip › Supplementary_Table_S2.pdf]

Supplementary Table S2.

Baseline preoperative CONUT category distribution and postoperative recovery according to sex

Variable	Male Normal (n=29)	Male Mild (n=44)	Male Moderate–Severe (n=18)	Female Normal (n=20)	Female Mild (n=27)	Female Moderate–Severe (n=12)	p-value*
**Overall complications, n (%)**	6 (20.7)	14 (31.8)	10 (55.6)	4 (20.0)	8 (29.6)	5 (41.7)	0.021
**Major complications (CD III–V), n (%)**	1 (3.4)	4 (9.1)	5 (27.8)	1 (5.0)	2 (7.4)	3 (25.0)	0.011
**Anastomotic leak, n (%)**	1 (3.4)	2 (4.5)	3 (16.7)	0 (0.0)	1 (3.7)	2 (16.7)	0.034
**Time to first flatus (days)**	3.2 ± 0.8	3.8 ± 1.0	4.6 ± 1.3	3.1 ± 0.9	3.7 ± 1.1	4.4 ± 1.2	<0.001
**Time to oral diet (days)**	5.1 ± 1.1	5.9 ± 1.4	7.2 ± 1.8	5.0 ± 1.2	5.8 ± 1.5	6.8 ± 1.7	<0.001
**Hospital stay (days)**	8.8 ± 2.5	10.8 ± 3.2	14.5 ± 4.6	9.0 ± 2.8	10.4 ± 3.1	13.6 ± 4.2	<0.001
**Delayed functional recovery, n (%)**	5 (17.2)	15 (34.1)	11 (61.1)	4 (20.0)	9 (33.3)	7 (58.3)	<0.001
**90-day mortality, n (%)**	0 (0.0)	1 (2.3)	2 (11.1)	0 (0.0)	0 (0.0)	1 (8.3)	0.048

Patients were categorized according to baseline preoperative CONUT score as normal nutritional status (0–1 points), mild immunonutritional impairment (2–4 points), and moderate–severe immunonutritional impairment (≥5 points). Postoperative recovery parameters, complication rates, and mortality outcomes were subsequently compared across sex-specific CONUT strata. Continuous variables are presented as mean ± standard deviation, whereas categorical variables are presented as number (percentage). P-values refer to comparisons across all six study groups. Higher baseline CONUT scores were consistently associated with less favorable postoperative recovery profiles in both male and female patients.
